# Deleterious Effects of Cold Air Inhalation on Coronary Physiological Indices in Patients With Obstructive Coronary Artery Disease

**DOI:** 10.1161/JAHA.118.008837

**Published:** 2018-07-12

**Authors:** Rupert P. Williams, Kaleab N. Asrress, Matthew Lumley, Satpal Arri, Tiffany Patterson, Howard Ellis, Vasiliki Manou‐Stathopoulou, Catherine Macfarlane, Shruthi Chandran, Kostantinos Moschonas, Pippa Oakeshott, Timothy Lockie, Amedeo Chiribiri, Brian Clapp, Divaka Perera, Sven Plein, Michael S. Marber, Simon R. Redwood

**Affiliations:** ^1^ Cardiovascular Division Rayne Institute St Thomas’ Hospital King's College London London United Kingdom; ^2^ Population Health Research Institute St George's University of London United Kingdom; ^3^ Leeds University Leeds Teaching Hospitals NHS Trust Leeds United Kingdom

**Keywords:** cold, coronary, coronary flow, coronary microvascular resistance, physiology, wave intensity analysis, Coronary Circulation, Hemodynamics, Ischemia, Physiology

## Abstract

**Background:**

Cold air inhalation during exercise increases cardiac mortality, but the pathophysiology is unclear. During cold and exercise, dual‐sensor intracoronary wires measured coronary microvascular resistance (MVR) and blood flow velocity (CBF), and cardiac magnetic resonance measured subendocardial perfusion.

**Methods and Results:**

Forty‐two patients (62±9 years) undergoing cardiac catheterization, 32 with obstructive coronary stenoses and 10 without, performed either (1) 5 minutes of cold air inhalation (5°F) or (2) two 5‐minute supine‐cycling periods: 1 at room temperature and 1 during cold air inhalation (5°F) (randomized order). We compared rest and peak stress MVR, CBF, and subendocardial perfusion measurements. In patients with unobstructed coronary arteries (n=10), cold air inhalation at rest *decreased* MVR by 6% (*P*=0.41), increasing CBF by 20% (*P*<0.01). However, in patients with obstructive stenoses (n=10), cold air inhalation at rest *increased* MVR by 17% (*P*<0.01), reducing CBF by 3% (*P*=0.85). Consequently, in patients with obstructive stenoses undergoing the cardiac magnetic resonance protocol (n=10), cold air inhalation reduced subendocardial perfusion (*P*<0.05). Only patients with obstructive stenoses performed this protocol (n=12). Cycling at room temperature decreased MVR by 29% (*P*<0.001) and increased CBF by 61% (*P*<0.001). However, cold air inhalation during cycling blunted these adaptations in MVR (*P*=0.12) and CBF (*P*<0.05), an effect attributable to defective early diastolic CBF acceleration (*P*<0.05) and associated with greater ST‐segment depression (*P*<0.05).

**Conclusions:**

In patients with obstructive coronary stenoses, cold air inhalation causes deleterious changes in MVR and CBF. These diminish or abolish the normal adaptations during exertion that ordinarily match myocardial blood supply to demand.

**Clinical Perspective**

**What Is New?**

- This study was performed to further our understanding of the pathophysiological effects of exertion in the cold.
- We used cold air inhalation as a physiological cold stressor and designed a study protocol enabling supine exercise to be performed on the cardiac catheter laboratory table during coronary angiography.
- Additionally, we used dual‐sensor pressure and flow intracoronary guidewires to enable continuous and simultaneous acquisition of coronary blood flow and microvascular resistance.
- We demonstrated that cold air inhalation during supine exercise severely blunted coronary vasodilation and the increase in coronary blood flow compared with supine exercise at room temperature, significantly increasing myocardial ischemia.

**What Are the Clinical Implications?**

- Our study demonstrates the potential hazardous mismatch between coronary flow and myocardial work caused by even brief periods of cold air inhalation with and without exercise, particularly in patients who have coronary artery disease and who have a reduced vasodilator reserve.
- Doctors should inform patients with obstructive coronary stenoses (1) to avoid exertion in cold air where possible, or at least to wrap up warmly (ideally involving coverage of the cheeks, mouth, and forehead); (2) that their exercise tolerance may be less in cold air; and (3) indoor warm‐up exercise before exertion in cold air might attenuate these deleterious effects.

## Introduction

Cold air inhalation during vigorous physical exertion, especially shoveling snow,^1^ results in significant increases in exertion‐related cardiac deaths.^2^ Cold air alone is associated with an increased incidence of myocardial infarction and cardiac mortality,^3^ independent of confounding factors such as respiratory illness.^3^ Exertion in the cold has also been shown to reduce time to angina, and time to electrocardiographic ST‐segment depression,^4^ compared with exertion at room temperature.

The pathophysiology of sudden cardiac death during vigorous exertion in cold air is unclear but likely to be multifactorial.^4^, ^5^, ^6^, ^7^ One of the triggering events may be the deleterious effect of cold air on coronary blood flow.^5^, ^8^ Patients with obstructive coronary stenoses have less coronary vasodilator reserve and maybe particularly vulnerable to such deleterious effects.^9^ Changes in coronary blood flow are principally mediated by changes in coronary microvascular resistance (MVR). However, to date no in vivo MVR measurements have been taken during cold air inhalation.

Dual‐sensor intracoronary guidewires now allow accurate continuous in vivo measurement of distal coronary artery pressure and Doppler‐flow velocity, permitting calculation of MVR.^10^ Continuous acquisition of these data also enables application of wave intensity analysis to identify the primary events driving coronary blood flow velocity (CBF) during each cardiac cycle, thereby examining the relationship between cardiac contraction and CBF. In addition, cardiac magnetic resonance (CMR) techniques now have the spatial resolution necessary to quantify transmural gradients of myocardial blood flow^11^, ^12^, ^13^ and subendocardial perfusion, the myocardial layer most vulnerable to ischemia.^14^

The aims of this descriptive study are as follows:

1. To determine the effect of the presence or absence of an obstructive coronary artery stenosis on the hemodynamic response (MVR, CBF, and wave intensity analysis) to a period of cold air inhalation at rest.
2. In patients with an obstructive coronary artery stenosis, to determine the additional effect of cold air inhalation during a period of exercise on the same hemodynamic variables, compared with exercise during room temperature as a control.

## Methods

The data, analytic methods, and study materials have been made available to other researchers for purposes of reproducing the results or replicating the procedure.^7^

**Figure 1:**
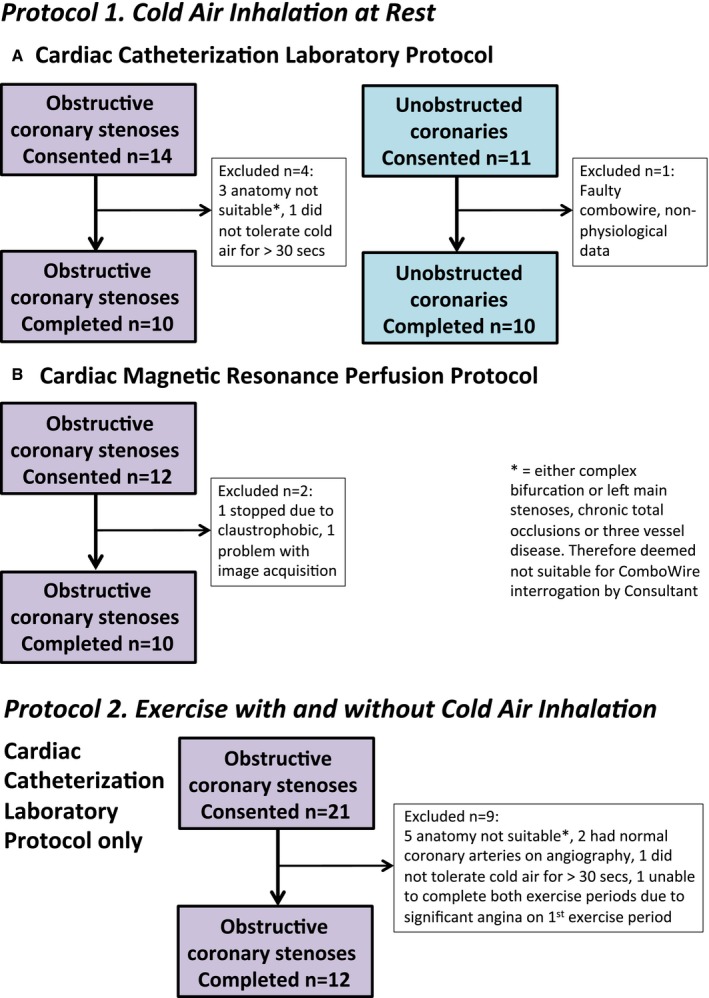
Consort diagram for flow of patients through the study. Of the 58 patients consented into study protocols, 42 successfully completed full protocols. No patients completed more than 1 protocol. (1) Cold air inhalation at rest: the effect of the presence or absence of an obstructive coronary artery stenosis on the hemodynamic response to a period of cold air inhalation at rest. (2) Exercise with and without cold air inhalation (in patients with obstructive coronary stenoses only): the additional effect of cold air inhalation during a period of exercise was assessed in the same variables, compared with exercise at room temperature as a control.

Between April 2013 and July 2015, we recruited 42 patients (62±9 years) from routine coronary angiography waiting lists, in 2 distinct groups (Figure 1):

1. Patients with obstructive coronary stenoses (n=32): defined by either a coronary artery stenosis >50% on quantitative coronary angiography^15^ or fractional flow reserve ≤0.8.^16^ These patients had typical anginal symptoms and multiple risk factors, or known obstructive coronary artery stenoses awaiting percutaneous coronary intervention.
2. Patients with unobstructed coronary arteries (n=10): defined by a coronary artery stenosis <30% on quantitative coronary angiography. The majority of these patients had atypical chest pain with some cardiac risk factors, and some had a mildly positive or equivocal exercise treadmill test or ischemia test.

Exclusion criteria for all groups were as follows: patients with 30% to 50% stenosis on quantitative coronary angiography; unstable symptoms; impaired left ventricular function; valvular heart disease; coronary artery bypass surgery; left main‐stem or complex bifurcation coronary artery stenoses; multivessel coronary disease; chronic total occlusions; severe comorbidities; severe chronic obstructive pulmonary disease or asthma (to minimize risk of bronchospasm with cold air inhalation); and an inability to undertake exercise and standard contraindications to CMR.

All vasoactive medications were stopped 48 hours before the procedure. Subjects gave written informed consent in accordance with the protocol approved by the institutional ethics review committee (REC 13/LO/0759). The study was registered with the National Institute for Health Research (NIHR) UK Clinical Research Network (UKCRN) portfolio database (ID 131491).

### Study Interventions

#### Cold air inhalation

Cold air was delivered via a loose‐fitting facemask attached to the patient's face for 5 minutes, at a fixed temperature of 5°F and a flow rate of 50 L/min (using an adapted cryotherapy device with a closed cooling circuit: Cryo6 Cold Air Device; Zimmer Medizin Systeme, Ulm, Germany). The cold air chilled the cheeks, nose, mouth, and laryngeal cold receptors.^5^

#### Exercise

An adapted supine ergometer (Ergosana, Schiller, Germany) was attached to the cardiac catheterization laboratory table.^17^ Exercise started at 50 W, with 20‐W increments every minute. It continued for 5 minutes unless the following occurred: severe angina, breathlessness, fatigue, or target heart rate achieved (220 beats per minute−age).

### Study Design

All studies were performed at room temperature (70°F). The potential psychological effect of wearing the facemask at baseline (without cold air inhalation) was assessed in all patients. Patients were randomly assigned to complete 1 of the 2 protocols (Figure 1), whereby baseline and peak stress measurements were acquired before and after *either*:

Protocol 1. Cold air inhalation at rest (n=30): 5 minutes of cold air inhalation at rest, in either an (1) invasive or (2) noninvasive protocol, as below:
  - Cardiac Catheterization Laboratory Protocol (invasive coronary flow velocity and pressure data): in patients with obstructed coronary stenoses (n=10) and patients with unobstructed coronary arteries (n=10).
  - CMR Protocol (subendocardial to subepicardial perfusion gradients): in a further separate group of patients with obstructive coronary stenoses (n=10).
  OR
Protocol 2. Exercise with and without cold air inhalation (n=12): Two 5‐minute supine‐cycling exercise periods: 1 at room temperature AND 1 during cold air inhalation. Only patients with obstructive coronary stenoses (n=12) were recruited to this study, and only the Cardiac Catheterization Laboratory Protocol was used. Exercise in room temperature was used as our control. The exercise periods were performed in randomized order to minimize potential warm‐up effects of prior exercise.^17^ There was a minimum of 15 minutes rest between exercise periods to allow hemodynamics to return to baseline.

### Study Protocols

#### Cardiac catheterization laboratory protocol

Patients were catheterized via the right radial or femoral artery with standard 6‐Fr coronary guide catheters (although radial arterial access was mandated in all patients undergoing exercise protocols) (Video S1, Figure S1). Patients received 300 mg of aspirin and 600 mg of clopidogrel orally, and 70 units/kg heparin intra‐arterially. A dual pressure and Doppler sensor‐tipped 0.014‐in intracoronary wire (ComboWire, Volcano Corp, San Diego, CA) was used to measure coronary pressure and Doppler‐flow velocity. Wire pressure was first equalized to that recorded by the fluid‐filled manometer and then the wire tip was advanced into the distal target coronary artery and manipulated until a stable and optimal Doppler‐flow velocity trace was obtained. Hemodynamic measurements were taken under resting conditions and continuously during each stressor.

#### CMR protocol

Cine and myocardial perfusion images were acquired on a 3‐T CMR scanner (Achieva Tx, Philips Medical Systems, Best, the Netherlands) at baseline and during peak cold air inhalation at rest (scan started 3 minutes after cold air inhalation commenced). High spatial resolution (1.2×1.2 mm) perfusion images were acquired in midsystole using a saturation recovery gradient echo method.^12^, ^13^ Further details are specified in Data S1.

### Hemodynamic Analyses

Aortic pressure, distal coronary artery pressure, and average peak Doppler‐flow velocity data were sampled at 200 Hz and stored on disk for off‐line analysis. Data were imported into custom software (Study Manager, Academic Medical Center, University of Amsterdam, the Netherlands), and 10 consecutive beats were extracted for analysis. Another custom‐made program, Cardiac Waves (Kings College London, UK), was then used to perform all further analyses. More detailed methods are specified in Figure S1.

*Average distal coronary artery Doppler‐flow velocity (CBF) and pressure (P*_*d*_ *)* were obtained from the ComboWire. MVR was calculated as (P_d_/CBF).^10^ “Minimal” MVR was also calculated as previously defined,^18^ through identification of the wave‐free period in diastole with wave intensity analysis when resistance is at its lowest. Minimal MVR therefore excludes systolic compressive resistance and better informs vascular resistance, and has been shown not to be influenced by coronary artery stenosis severity.^18^ Trans‐stenotic pressure gradient (aortic pressure (P_a_)−P_d_), P_d_/P_a_ ratio, and epicardial stenosis resistance (P_a_−P_d_/average peak velocity)^19^ were also calculated as measures of stenosis severity. Epicardial stenosis resistance, defined as the ratio of the pressure drop across the stenosis to distal coronary flow velocity, normalizes the pressure drop for the magnitude of epicardial coronary flow velocity at which it was obtained, providing a more objective assessment of hemodynamic stenosis severity. Moreover, these data combined allow the selective evaluation of epicardial and MVR to coronary blood flow.^20^

**Figure 2:**
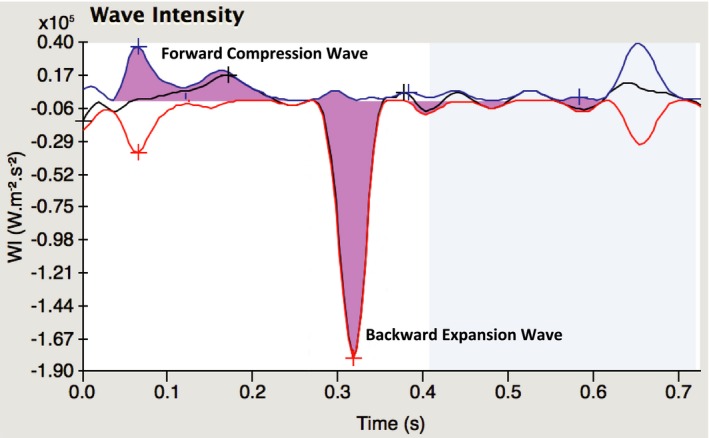
Coronary wave intensity (WIA) profile at rest. This figure is a typical example of a WIA profile obtained at rest from a patient with unobstructed coronary arteries. The 2 shaded areas represent the forward compression and backward expansion wave intensities.

*Coronary wave intensity analysis:* Continuous acquisition of simultaneous changes in distal CBF and pressure also allows insight into the phasic components of forces driving CBF. Net coronary wave intensity (dI) was calculated as the product of the derivatives of ensemble‐averaged distal coronary pressure (P_d_) and Doppler‐flow velocity (CBF), so that: dI=dP_d_/dt×dCBF/dt.^21^ We calculated, as previously described,^22^, ^23^ the 2 dominant waves that drive coronary perfusion (Figure 2): (1) The forward compression wave, a systolic acceleratory wave generated by sudden increases in aortic pressure at the inlet during ventricular ejection. (2) The backward expansion wave, an early diastolic acceleratory wave generated by microcirculatory suction from the rapid reduction in left ventricular pressure during ventricular relaxation (relieving compression of intramyocardial vessels).^22^, ^23^

*Aortic pulse wave analysis* (from central arterial pressure waveforms): Augmentation index (a central measure of afterload), rate pressure product (a measure of myocardial oxygen consumption),^17^ and diastolic time fraction were calculated as previously described.^17^

*Myocardial perfusion analysis:* Myocardial perfusion CMR images were analyzed using dedicated software (EasyScil prototyping [Philips Medical Systems, Best, the Netherlands] and Matlaboratory [MathWorks Inc, Natick, MA]). Absolute myocardial blood flow and subendocardial to subepicardial perfusion gradients were calculated as previously described.^12^, ^13^ These maximize the high spatial and temporal resolution of the CMR perfusion sequence, and present the distribution of myocardial blood flow between the subendocardium and subepicardium over time^12^, ^13^ (Figure S2). An increased gradient suggests subendocardial ischemia, using prospectively established thresholds.^12^ Further details are specified in Data S1.

### Statistical Methods

Continuous variables were tested for normality using the Shapiro–Wilk test and presented as mean±SEM when data were normally distributed or as median with interquartile range.

All data were analyzed (1) at baseline and (2) during peak stress. Continuous data were examined every 30 s throughout the invasive protocol and peak stress was defined as the data set with the highest rate pressure product. A repeated‐measures 2‐way ANOVA was performed with matched values stacked into a subcolumn. To assess differences between rest and stress data, Sidak's multiple comparison test was then performed (confidence intervals and significance were computed, and an adjusted significance threshold of *P*≤0.05 was used for these comparisons). Between‐group differences in absolute change were assessed using paired or unpaired *t* tests (continuous and normal data) or Mann–Whitney *U* test or Wilcoxon signed‐rank test (non‐normal continuous data) as appropriate. A 2‐tailed test for significance was performed in all of the analyses.

Investigators performing data analyses were blinded to all clinical data. Statistical analyses were performed using GraphPad Prism 6.0 (GraphPad, San Diego, CA). *P* values of ≤0.05 were considered statistically significant.

## Results

### Study Population

Of the 58 patients consented into the study across the different protocols, Figure 1 shows that 42 successfully completed a full protocol (32 completed a cardiac catheterization laboratory protocol and 10 completed a CMR protocol). Sixteen patients were excluded: 14 from the cardiac catheterization laboratory protocol and 2 from the CMR protocol. In addition, 6 data sets were excluded from coronary hemodynamic and wave intensity analyses because of poor quality Doppler‐flow waveforms. Table 1 shows patient characteristics. Wearing the facemask (without cold air inhalation) at baseline did not cause significant changes to any variables.

**Table 1:** Patient Characteristics

Stressor (s)	Cold Air at Rest: Cath Lab Study	Cold Air at Rest: Cath Lab Study	Cold Air at Rest: CMR Study	Exercise With and Without Cold Air: Cath Lab Study
Patient Demographic	Unobstructed Coronaries	Obstructive Stenoses	Obstructive Stenoses	Obstructive Stenoses
Number of patients	10	10	10	12
Age, y	67.3±2.9	64.6±2.6	59.9±2.2	58.8±2.7
Male	6 (60%)	8 (80%)	8 (80%)	12 (100%)
BMI, kg/m^2^	29.1±1.3	33.9±2.3	29.9±1.7	30.2±2.0
Medical history				
Hypertension	9 (90%)	9 (90%)	7 (70%)	9 (69%)
Diabetes mellitus	2 (20%)	4 (40%)	4 (40%)	2 (15%)
Hypercholesterolemia	9 (90%)	9 (90%)	8 (80%)	11 (85%)
Smokers	3 (30%)	6 (60%)	7 (70%)	7 (54%)
Procedural details				
Stenosis of target lesion, %	19.9±2.4	76.5±4.1	72.9±3.8	68.6±4.7
Target vessel (LAD/Cx/RCA)	7/2/1	7/1/2	3/4/3	10/2/1

Values are mean±SEM or %. BMI indicates body mass index; Cath lab, cardiac catheterization laboratory; CMR, cardiac magnetic resonance; Cx, circumflex artery; LAD, left anterior descending artery; Obstructive stenoses, patients with obstructive coronary stenoses; RCA, right coronary artery; unobstructed coronaries, patients with unobstructed coronary arteries.

### Coronary MVR and CBF

#### Cold air inhalation at rest

**Figure 3:**
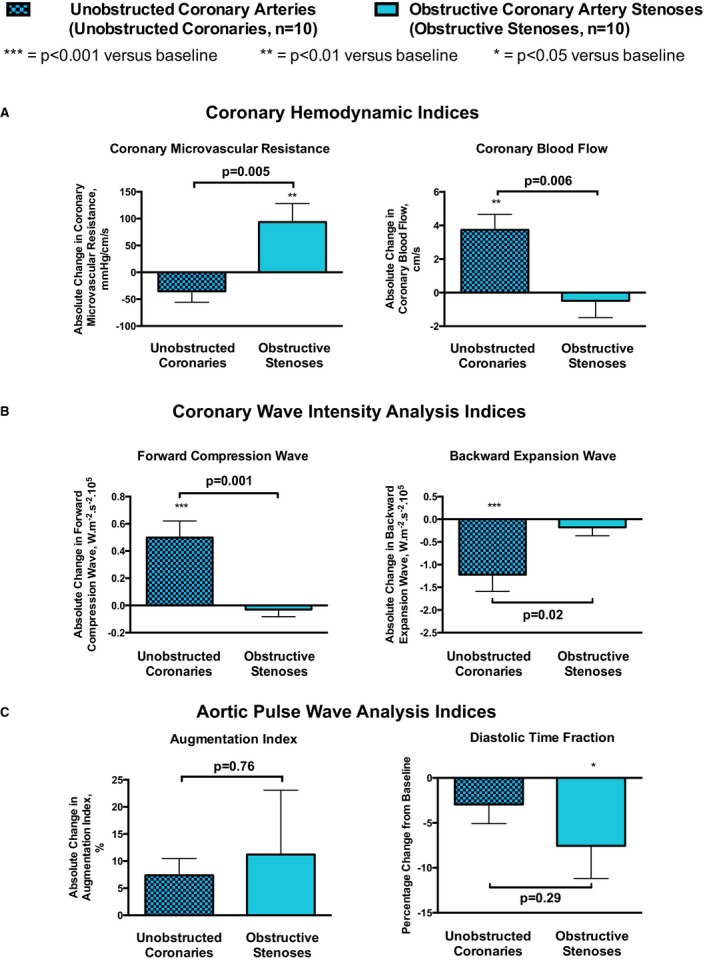
Cold air inhalation at rest. Change from baseline in patients with and without obstructive coronary stenoses, with the results subcategorized as per the following: (A) coronary hemodynamic results, (B) coronary wave intensity analysis results, and (C) aortic pulse wave analysis results.

**Figure 4:**
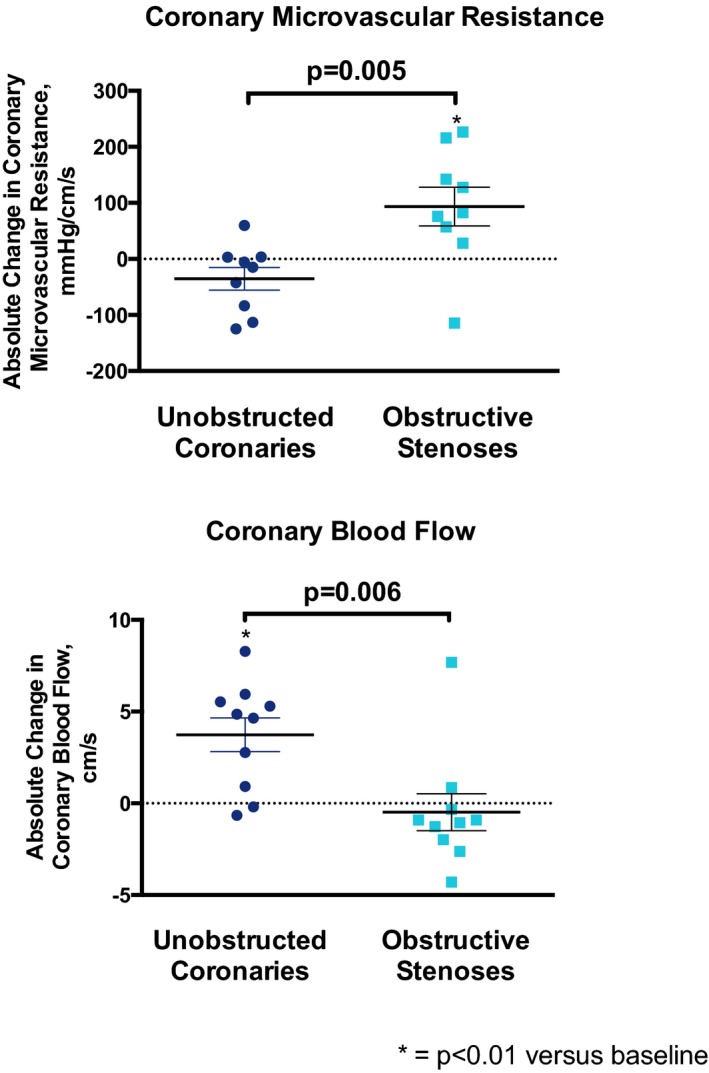
Individual responses to cold air inhalation at rest. The individual responses to cold air inhalation at rest demonstrate reasonable homogeneity across both groups, with the exception of 1 patient in each group. On further scrutiny of these individual patients, age appears to be a significant confounder. The patient with normal coronary arteries with an increase in microvascular resistance was the oldest in the study cohort, aged 80 years. It is well established that diastolic dysfunction increases with age, and this was likely sufficient to provoke inefficient ventricular ejection and relaxation despite epicardial patency. The patient who had an obstructive coronary stenosis and who managed to reduce microvascular resistance was the youngest in the study cohort, aged 50 years. Hence, despite a significant stenosis, better endothelial function combined with greater ventricular energetic reserve likely provided sufficient stimuli to maintain vasodilatation despite α‐1‐adrenoceptor‐mediated vasoconstriction.

At baseline before cold air was applied, patients with obstructive stenoses had significantly lower minimal MVR than patients with unobstructed coronary arteries (*P*<0.05) (Figures 3A and 4, Table 2). In patients with unobstructed coronary arteries, cold air inhalation at rest caused 6% and 8% *reductions* in MVR (*P*=0.41) and minimal MVR (*P*=0.27), respectively. This enabled a 20% increase in CBF (*P*<0.01). However, in patients with obstructive coronary stenoses, there were deleterious 17% and 24% *increases* in MVR (*P*<0.01) and minimal MVR (*P*<0.01), respectively, resulting in a 3% reduction in CBF (*P*=0.85). This resulted in between‐group differences in absolute change for both MVR (*P*=0.005) and CBF (*P*=0.006).

**Table 2:** Catheter Laboratory Coronary and Aortic Hemodynamics and Wave Intensity Analysis

Condition	Cold Air Inhalation at Rest				Exercise at Room Temp		Exercise During Cold Air	
Patient Demographic	Unobstructed Coronaries		Obstructive Stenoses		Obstructive Stenoses		Obstructive Stenoses	
	Baseline	Peak	Baseline	Peak	Baseline	Peak	Baseline	Peak
Stenosis severity								
P_d_ (mean)/P_a_ (mean) ratio	0.96±0.01	0.97±0.01	0.83±0.08	0.83±0.08	0.88±0.05	0.82±0.05^a^	0.90±0.04	0.87±0.05^a^, ^b^
Trans‐stenotic gradient, mm Hg	4.2±0.9	3.4±1.3	15.9±7.2	19.0±9.3	14.2±5.9	24.8±6.6^a^	12.1±5.8	18.9±6.7^a^, ^b^
Epicardial stenosis resistance, mm Hg/cm per s	0.26±0.06	0.18±0.07	0.35±0.06	0.41±0.08	0.32±0.08	0.58±0.11^a^	0.33±0.08	0.46±0.09
Coronary hemodynamics								
Mean distal pressure, mm Hg	99.1±3.7	114.5±3.7^a^	87.7±10.9	102.6±11.8^a^	96.6±4.2	111.1±6.7^a^	100.6±3.8	121.8±7.8^a^
Coronary blood flow velocity, cm/s	18.1±1.7	21.8±1.9^a^	17.3±2.1	16.8±2.2	19.9±2.7	32.1±4.3^a^	20.5±2.1	27.7±3.0
Microvascular resistance, mm Hg/cm per s	612±54	576±48	550±44	643±54^a^	563±66	397±43^a^	550±42	476±45
Min. MVR, mm Hg/cm per s	449±43	415±35	317±28	394±28^a^	373±47	221±33^a^	328±33	288±40^b^
Coronary wave intensity analysis								
Forward compression wave, J·m^−2^·s^−2^·10^5^	0.52±0.09	1.03±0.15^a^	0.59±0.09	0.56±0.12^c^	0.59±0.13	1.82±0.48^a^	0.56±0.10	1.56±0.42^a^
Backward expansion wave, J·m^−2^·s^−2^·10^5^	1.74±0.29	2.98±0.36^a^	1.53±0.30	1.71±0.30^c^	1.91±0.21	6.47±1.34^a^	2.05±0.31	4.09±0.81^b^
Aortic hemodynamics								
Heart rate, bpm	72.5±4.6	76.3±3.6	74.1±3.4	75.1±2.9	79.4±3.8	121.9±5.5^a^	82.2±4.0	127.6±5.3^a^
Mean aortic pressure, mm Hg	103.3±3.4	117.9±3.7^a^	103.6±6.6	121.6±5.8^a^	107.0±3.0	131.7±4.2^a^	110.0±4.1	135.6±4.8^a^
Systolic aortic pressure, mm Hg	143.5±5.0	164.8±8.1^a^	139.4±10.1	166.6±8.1^a^	138.5±3.7	170.1±6.3^a^	141.6±4.6	181.5±7.0^a^
Diastolic aortic pressure, mm Hg	72.8±3.4	84.3±2.7^a^	78.0±4.7	87.4±5.0^a^	81.4±2.2	95.9±2.3^a^	82.7±2.7	97.5±3.1^a^
Rate pressure product, mm Hg·min^−1^·10^4^	10.4±0.8	12.4±0.6^a^	10.4±0.9	12.5±0.8^a^	10.9±0.7	20.8±1.2^a^	11.5±0.4	23.1±1.1^a^
Augmentation index, %	37.8±6.2	45.8±5.2	38.7±6.0	49.9±10.4	51.1±10.4	27.2±6.1^a^	48.6±11.0	46.1±11.0
Diastolic time fraction	0.59±0.02	0.57±0.01	0.58±0.02	0.54±0.02^a^	0.52±0.03	0.41±0.03^a^	0.53±0.02	0.35±0.02^a^,^b^

Values are mean±SEM. Baseline indicates before application of cold air; Min. MVR, minimal MVR; obstructive stenoses, patients with obstructive coronary artery stenoses; P_a_ (mean), mean aortic pressure; P_d_ (mean), mean distal pressure; temp, temperature; U, coronary blood flow velocity; unobstructed coronaries, patients with unobstructed coronary arteries.

a *P*<0.05 vs respective baseline.

b *P*<0.05 vs peak exercise room temp.

c *P*<0.05 vs peak normal coronary arteries.

#### Exercise with and without cold air inhalation in patients with obstructive stenoses

**Figure 5:**
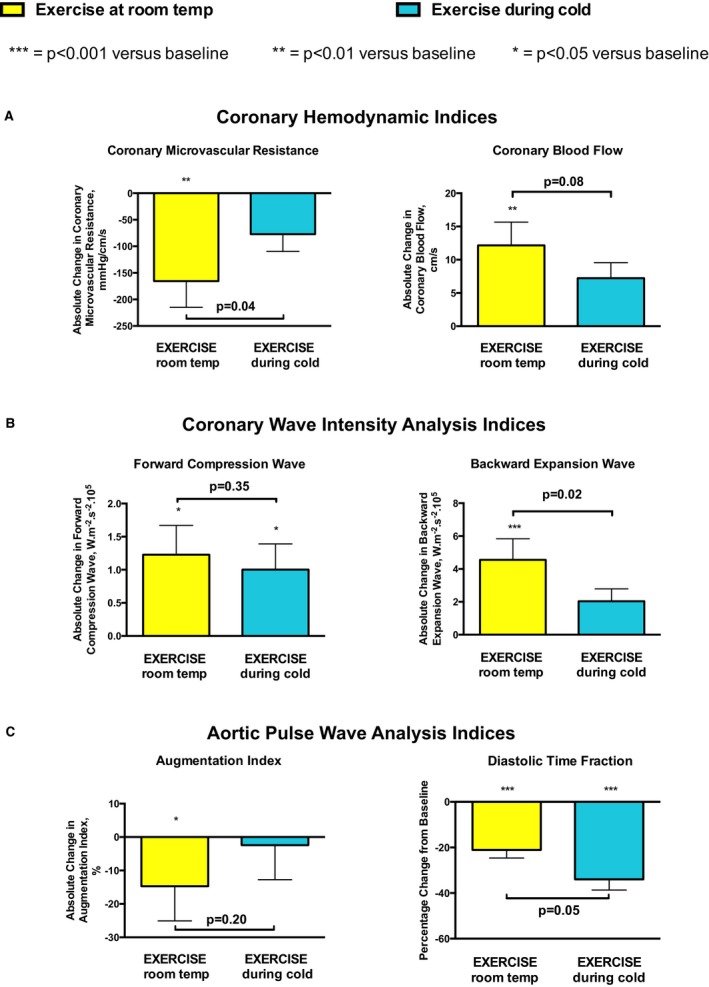
Exercise with and without cold air inhalation. Change from baseline in patients with obstructive coronary stenoses, with the results subcategorized as per the following: (A) coronary hemodynamic results, (B) coronary wave intensity analysis results, and (C) aortic pulse wave analysis results.

**Figure 6:**
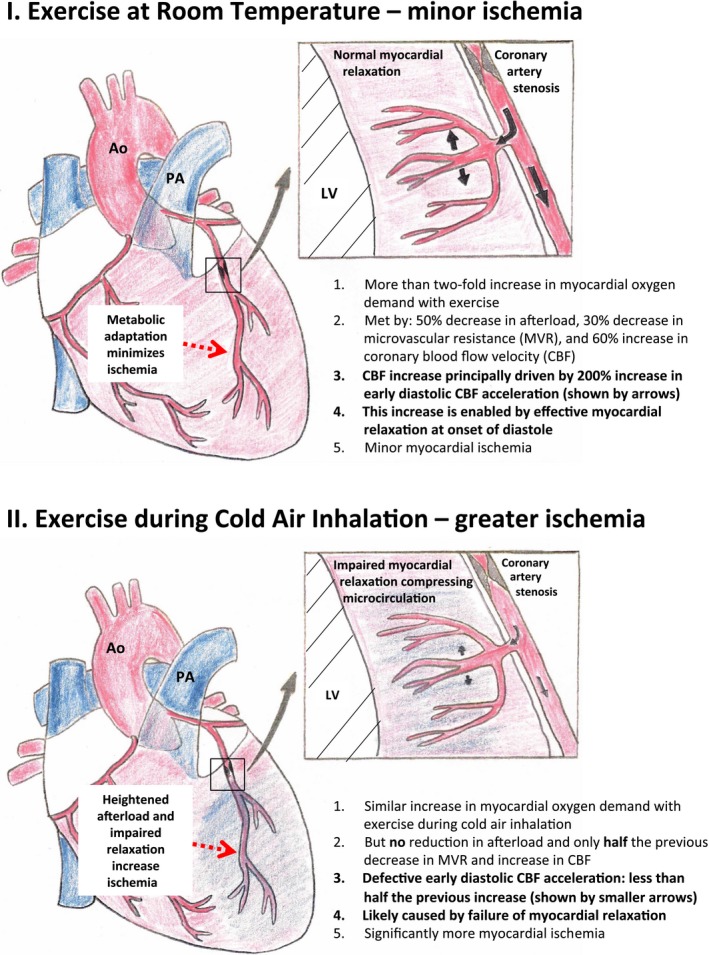
Pathological effects of cold air during exercise in 12 patients with obstructive coronary stenoses. Ao indicates aorta; CBF, coronary blood flow velocity; LV, left ventricle; MVR, microvascular resistance; PA, pulmonary artery.

During exercise at room temperature, MVR and minimal MVR decreased by 29% (*P*<0.001) and 41% (*P*<0.001), respectively, associated with a 61% increase in CBF (*P*<0.001) (Figures 5A and 6, Table 2). However, MVR and minimal MVR decreased by only 13% (*P*=0.12) and 12% (*P*=0.48), respectively, during exercise in cold air inhalation, associated with a smaller (35%) increase in CBF (*P*<0.05). Paired group analysis also demonstrated that absolute reductions in MVR and minimal MVR during exercise with cold air inhalation were significantly smaller than during exercise in room temperature (*P*=0.04, Figure 5A) and (*P*<0.01), respectively.

### Coronary Wave Intensity Analysis

#### Cold air inhalation at rest

In patients with unobstructed coronary arteries, cold air inhalation increased the energy of the 2 dominant waves that drive coronary perfusion: the forward compression wave by 98% (*P*<0.001) and the backward expansion wave by 71% (*P*=0.001) (Figure 3B, Table 2). However, in patients with obstructive coronary stenoses, cold air inhalation actually caused a 5% reduction in the magnitude of the forward compression wave (*P*=0.93) and abrogated the backward expansion wave response to a 12% increase (*P*=0.79). This resulted in between‐group differences in absolute change for both the forward compression wave (*P*=0.001) and the backward expansion wave (*P*=0.02) (Figure 3B).

#### Exercise with and without cold air inhalation in patients with obstructive stenoses

Exercise at room temperature resulted in profound increases to the forward compression and backward expansion waves of 208% (*P*<0.05) and 239% (*P*<0.001), respectively (Figures 5B and 6, Table 2). However, during exercise in cold air inhalation, the backward expansion wave only increased by 95% (*P*=0.13). Paired group analysis also demonstrated that the absolute increases in the backward expansion wave during exercise with versus without cold air inhalation were significantly different (*P*=0.02, Figures 5B and 6). In contrast, there remained a 178% increase in the forward compression wave (*P*<0.05) during exercise in cold air inhalation.

### Aortic Pulse Wave Analysis

#### Cold air inhalation at rest

Increases in rate pressure product and augmentation index during cold air inhalation were similar for patients with and without obstructive coronary stenoses (Figure 3C, Table 2). However, diastolic time fraction was deleteriously decreased in patients with obstructive coronary stenoses (*P*=0.04), but not in patients with unobstructed coronary arteries (*P*=0.49).

#### Exercise with and without cold air inhalation in patients with obstructive stenoses

Increases in rate pressure product were greater during exercise with versus without cold air inhalation, although this did not reach significance (*P*=0.06) (Figures 5C and 6, Table 2). Exercise at room temperature decreased augmentation index by 47% (*P*<0.05), but the addition of cold air inhalation during exercise abolished this adaptive reduction to only 5% (*P*=0.95). The diastolic time fraction reduced during exercise with and without cold air inhalation (by 31% and 21%, respectively, both *P*<0.001). However, overall, the addition of cold air inhalation during exercise caused a further deleterious absolute reduction in diastolic time fraction compared with exercise in room temperature (*P*=0.05).

### Electrocardiographic ST‐Segment Depression

Apart from 1 patient with an obstructive stenosis who withdrew after developing angina, cold air inhalation at rest did not result in any reported angina or measurable ST‐segment depression. Maximal ST‐segment depression was greater during exercise with cold air inhalation versus without (0.17±0.02 mV versus 0.12±0.02 mV, *P*=0.02). The maximum tolerated exercise duration was 313±15 s versus 337±24 s for exercise with versus without cold air (*P*=0.13), with similar workloads (116±7 W versus 115±7 W, respectively, *P*=0.84. This is equivalent to between 6.5 and 9 metabolic equivalents or stage 2‐3 Bruce protocol).

### Stenosis Severity

Measures of stenosis severity did not significantly increase in either group during cold air inhalation at rest (Table 2), although there was a trend (*P*=0.11) towards reduced epicardial stenosis resistance in patients with unobstructed coronary arteries and increased epicardial stenosis resistance in patients with obstructive stenoses. During exercise at room temperature, there were significant increases in trans‐stenotic gradient (*P*<0.001), P_d_/P_a_ ratio (*P*<0.01), and epicardial stenosis resistance (*P*<0.01). Unexpectedly during exercise in cold air, there were smaller increases seen in these parameters, and significantly lower peak trans‐stenotic gradient and P_d_/P_a_ ratio (*P*=0.02 and <0.01, respectively).

### CMR Analysis

Ten separate patients with obstructive coronary stenoses underwent the CMR protocol (Figure 2, Table 1). Cold air inhalation at rest caused significant deleterious increases in subendocardial to subepicardial transmural perfusion gradients from baseline, suggesting inadequate subendocardial perfusion (Figure S3). In keeping with invasive CBF data, there was no associated substantial increase in overall myocardial blood flow during cold air inhalation (0.92±0.14 mL/g per minute versus 1.07±0.11 mL/g per minute, *P*=0.19).

## Discussion

For the first time, we have combined intracoronary measurements of pressure and Doppler‐flow during cardiac catheterization to understand the pathophysiology of cold air inhalation during exercise. These measurements show that in patients with obstructive coronary stenoses, cold disrupts the fundamental physiological adaptations that ensure myocardial blood supply matches demand during exercise. In these patients, cold air inhalation prevented the decrease in myocardial MVR needed to increase blood flow during exercise, while at the same time increasing cardiac work through heightened afterload (Figure 6). High‐resolution myocardial perfusion CMR demonstrated that the increases in myocardial MVR and cardiac afterload manifested as a cold‐induced deficit in subendocardial perfusion. These deleterious changes render the myocardium vulnerable to ischemia, infarction, and potentially fatal cardiac arrhythmias.

The use of wave intensity analysis, unlike those of previous studies,^24^, ^25^ has allowed us to examine the events within a single cardiac cycle that result in the mismatch between myocardial blood flow and demand. Our most striking finding was that in patients with obstructive coronary stenoses, cold air during exercise abrogated the adaptive 200% increase in the backward expansion wave that accompanied exercise in room temperature (Figure 6). This microcirculatory “suction” wave results from the release of external compressive forces on resistance vessels during ventricular relaxation,^23^ which in turn decreases MVR. This wave is the largest magnitude wave in the cardiac cycle and enables early diastolic coronary flow acceleration following aortic valve closure.^23^

### Exercise in Room Temperature in Patients With Obstructive Stenoses

The mechanism of ventricular relaxation during exercise in room temperature deserves discussion. Despite increased myocardial oxygen demand and reduced diastolic time fraction,^17^ ischemia is minimized because of parallel adaptive reductions in afterload and MVR of 50% and 30%, respectively. The acute reduction in afterload enables a relatively efficient ejection time that is associated with enhanced ventricular relaxation,^26^ and in our study was evidenced by the 200% increase in the backward expansion wave. Ventricular relaxation during exercise may also be directly underpinned by efficient calcium reuptake via ß‐adrenoceptor‐mediated protein kinase A activation.^27^ Enhanced ventricular relaxation reduces microvascular compression,^28^ which helps lower MVR. Despite vasoconstriction of the epicardial stenosed segment during exercise^29^ (evidenced by increased stenosis severity in our study), parallel intense vasodilatation of resistance vessels during exercise^30^ overcomes this, resulting in further adaptive reduction of MVR.

### Cold Air During Exercise in Patients With Obstructive Stenoses

By contrast, ventricular relaxation is impaired with cold air during exercise (Figure 6). The adaptive reduction in afterload during exercise at room temperature is abolished: cold air caused a sufficiently potent sympathetic vasoconstrictive stimulus on the peripheral vasculature to offset the adaptive effects of dynamic exercise. This resulted in an additional pressure load on the ventricle, increasing relative ejection time and impairing ventricular relaxation.^31^ Ventricular relaxation may also be further impaired by relative autonomic conflict caused by parasympathetic activation of facial trigeminal receptors during cold air inhalation^32^: this would dampen ß‐adrenoceptor‐mediated protein kinase A activation mentioned in the paragraph above. Furthermore, we observed a significantly exaggerated reduction in the diastolic time fraction during exercise with cold air. This is an important additional finding, because subendocardial blood flow has been shown to be dependent on diastolic time fraction, independently of heart rate.^14^ Parallel to the adverse effect on afterload, cold air during exercise also halved the adaptive reduction in MVR during exercise at room temperature. This is likely because of a combination of heightened α‐1 adrenoceptor‐mediated vasoconstriction exhausting the vasodilator reserve, systolic myocardial compression of resistance vessels because of increased ejection time, and impaired ventricular relaxation. These adverse effects may also be self‐propagating, with increased myocardial ischemia further impeding ventricular relaxation via dysregulation of myocardial contractile machinery.

Unexpectedly, our measures of stenosis severity increased to a lesser extent during exercise in cold air versus exercise in room temperature (Table 2). This was seen in combination with a significantly higher peak minimal MVR during exercise in cold air versus exercise in room temperature. These data suggest that cold air during exercise results in relatively less epicardial resistance to coronary blood flow, but significantly more MVR to coronary blood flow. Therefore, these findings suggest that cold air–induced impaired vasodilatory capacity during exercise predominates at the arteriolar level, rather than the stenosed epicardial segment.

### Cold Air Inhalation at Rest in Patients With and Without Obstructive Stenoses

#### MVR and coronary blood flow

Despite a similar increase in cardiac afterload to cold air inhalation, MVR *decreased* in patients with unobstructed coronary arteries but *increased* in those with obstructive coronary stenoses. While vasodilatation of small arteries and arterioles may prevail over α‐1‐adrenoceptor‐mediated vasoconstriction in patients with unobstructed coronary arteries,^33^ patients with obstructive coronary stenoses are reliant on constant metabolic vasodilation of arterioles,^9^ evidenced by significantly lower minimal MVR at rest. These arterioles are therefore unable to compensate for vasoconstriction of small arteries in response to cold, resulting in increased MVR. In addition, these patients may have greater endothelial dysfunction and adverse structural remodeling of arterioles,^34^ resulting in a more potent vasoconstrictor response to cold air. This vasoconstrictor response predominantly affects the resistance vessels, not the stenosed epicardial artery, because we observed no significant increases in stenosis severity in either group (Table 2).

#### Wave intensity analysis

An interesting finding was the disparate response of the wave intensities to cold in patients with and without obstructive coronary stenoses. Patients with unobstructed coronary arteries significantly increased the forward compression and backward expansion waves during cold air inhalation. This reflects rapid ventricular ejection and surge in left ventricular pressure, and is associated with efficient ventricular relaxation.^26^ However, patients with obstructive coronary stenoses likely have a reduced ventricular energetic reserve,^35^ which when combined with significant resistance vessel vasoconstriction predisposes the efficiency of ventricular ejection and relaxation to be compromised in the face of acutely increased afterload.^31^ In addition, downregulation of ß‐adrenoceptors following chronic sympathetic stimulation^36^ in patients with obstructive stenoses may also dampen the inotropic and chronotropic response to cold air. We found that the heart rate increase to cold air in patients with unobstructed coronary arteries was more than 3 times the magnitude than patients with obstructed coronary arteries. Therefore, at peak stress these patients were unable to increase either the forward compression or backward expansion wave.

### Comparison With Other Studies

No previous studies have measured MVR, or applied wave intensity analysis or CMR perfusion techniques in response to cold air inhalation at rest or during exercise. Most studies for convenience used a “cold pressor test” at rest, whereby a limb is immersed into a bucket of iced water for 1 minute. However, this may not be physiologically relevant to exercising in cold air. In line with our findings, previous authors have found increases in MVR following a cold pressor test at rest using coronary sinus thermodilution in patients with coronary artery disease.^24^, ^25^ This increase in MVR has been previously shown to result from vasoconstriction predominating at the arteriolar level, as the MVR increase was out of proportion to the small epicardial vasoconstrictor response demonstrated with quantitative coronary angiography.^37^ Also in response to cold pressor test in patients with coronary “luminal irregularities,” CBF measured with intracoronary Doppler increased by a similar magnitude to that observed in our patients with unobstructed coronary arteries.^8^ Literature investigating cold air during dynamic exercise is very sparse, with 1 invasive study demonstrating slightly smaller reductions in MVR during exercise with simultaneous cold pressor test, versus during exercise alone.^25^

### Mechanism of Increased Cardiac Mortality Caused by Cold Air During Exercise

Although the mechanism remains unclear, our study demonstrated significantly greater myocardial ischemia during exercise with cold air inhalation through a facemask versus exercise at room temperature. The burden of ischemia during exercise in the cold will likely be even greater with more skin exposure to cold, and/or a longer duration of cold exposure. Observations from long‐distance running races suggest that ischemia burden alone may be sufficient to cause ventricular dysrhythmias and cardiac death.^38^ Moreover, cold stress may directly provoke arrhythmic cardiac death, through data demonstrating increased frequency of ventricular dysrhythmias during cold temperatures in patients with implanted cardioverter‐defibrillators.^39^ Additionally, cold air inhalation may increase the likelihood of a plaque rupture event. Notably, in 9 patients presenting with acute myocardial infarction after shoveling snow, all had angiographic evidence of plaque rupture.^1^

Unexpectedly, in our study we noted a smaller increase in trans‐stenotic gradient during exercise in cold air versus room temperature. This would counterintuitively protect the stenosis against a shear stress and a plaque rupture event! However, this was associated with higher mean distal arterial pressure during exercise in cold air and potentially greater circumferential wall stress, arguably a more important predictor of plaque rupture.^40^, ^41^ Shoveling snow and skiing involve isometric exercise in addition to dynamic exercise that will likely further increase myocardial oxygen demand and afterload.

### Clinical Implications

These findings could change clinical practice. Doctors should inform patients with obstructive coronary stenoses about the following: (1) to avoid sudden vigorous exertion, as asymptomatic ischemia can manifest before angina occurs; (2) to avoid exertion in cold air where possible, or at least to wrap up warmly (ideally involving coverage of the cheeks, mouth, and forehead); (3) that their exercise tolerance may be less in cold air; (4) indoor warm‐up exercise before exertion in cold air might attenuate these deleterious effects (by augmenting the reduction in MVR and increase in backward expansion wave intensity during the second period of exertion^17^). In addition, angiotensin‐converting enzyme inhibitors may attenuate coronary sympathetic vasoconstriction of resistance vessels,^42^ and glyceryl trinitrate may enhance diastolic ventricular relaxation,^43^ which may significantly reduce ischemia burden and possibly mortality with cold air.^44^

Novel therapeutic approaches to preventing myocardial ischemia induced by cold air may attempt to preserve ventricular relaxation, which is determined by the clearance of the cytosolic calcium transient and the sensitivity of the myofilaments to calcium. Both these processes are thought to contribute to heart failure with preserved ejection and are the subject of novel therapeutic intervention trials to influence them directly^45^, ^46^ or indirectly.^47^

### Limitations

Our study has several important limitations. First, similar to other physiological studies investigating the effects of cold,^5^, ^24^, ^25^ the sample size was small, which limits extrapolation. Because this is a hypothesis‐generating descriptive study, it is difficult to differentiate 1 association in a hemodynamic variable from another and we did not correct for multiple comparisons. Participants were predominantly male, and findings may not apply to females. Six ComboWire data sets were excluded from coronary hemodynamic and wave intensity analyses because of poor‐quality traces. However, given the relatively homogeneous physiological responses in all variables, we believe our data are generally representative of our entire patient cohort.

Another weakness is the lack of a direct measure of ventricular relaxation or plasma catecholamines, although the latter have been measured before in response to cold.^48^, ^49^ Further studies including intracoronary phentolamine and adenosine would be useful. We did not measure epicardial artery diameter changes during exercise because of safety concerns: Our protocol mandated removal of the guiding catheter away from the coronary ostium before starting exercise to reduce the risk of iatrogenic coronary artery dissection. Our control group of patients with unobstructed coronary arteries likely had endothelial dysfunction and impaired coronary vasomotion, because they were being investigated for chest pain with coronary angiography. However, this study was primarily designed to assess the effect of an obstructive coronary stenosis, and therefore our group classification remains relevant. Although we did not examine the effect of subjective cold air intolerance on the hemodynamic response to cold, cold tolerance was similar between groups. Finally, measurements of left ventricular pressure volume loops were not included in this exploratory study. Further studies might undertake simultaneous pressure volume loops and coronary wave intensity analysis.

## Conclusions

In patients with obstructive coronary stenoses, cold air inhalation during exercise severely blunts the adaptive reductions in afterload and MVR that usually occur during exercise at room temperature. More detailed scrutiny by wave intensity analysis suggests that this is the result of a failure of coronary blood flow acceleration at the onset of diastole. In addition to the avoidance of exercising in cold air, this deficit may be amenable in the future to pharmacological manipulation.

## Sources of Funding

This study received support for the design and conduct of the study; collection, management, analysis, and interpretation of the data; preparation and review of the article; and decision to submit the article for publication. British Heart Foundation Fellowships to Williams (FS/11/90/29087), Asrress (FS/11/43/28760), Plein (FS/10/62/28409) and award RE/08/003, the Engineering and Physical Sciences Research Council, the Wellcome Trust (088641/Z/09/Z) and the UK Department of Health through the National Institute for Health Research Biomedical Research Centre award to Guy's & St Thomas’ National Health Service Foundation Trust.)

## Disclosures

None.

## Supporting information


**Data S1.** Supplemental methods.
**Figure S1.** Cardiac catheterization laboratory protocol setup.
**Figure S2.** Transmural perfusion gradients with CMR perfusion scans.
**Figure S3.** Transmural perfusion gradients during cold air inhalation in patients with significant coronary artery disease.

**Video S1.** Cardiac catheterization laboratory protocol setup.
