# Supplementary material for: Deleterious Effects of Cold Air Inhalation on Coronary Physiological Indices in Patients With Obstructive Coronary Artery Disease
Source: J Am Heart Assoc. 2018 Jul 12;7(14):e008837. doi: 10.1161/JAHA.118.008837 (PMC6064824; doi:10.1161/JAHA.118.008837)
Supplement: Supplementary file 1 — Data S1. Supplemental methods. Figure S1. Cardiac catheterization laboratory protocol setup. Figure S2. Transmural perfusion gradients with CMR perfusion scans. Figure S3. Transmural perfusion gradients during cold air inhalation in patients with significant coronary artery disease. [file JAH3-7-e008837-s001.pdf]

Supplemental Material

Data S1.

Supplemental Methods

Cardiac Magnetic Resonance (CMR) Myocardial Perfusion Sequence

Myocardial perfusion CMR was performed using dual bolus injection of 0.0075/0.075mmol/Kg of Gadolinium diethyl-enetriaminepentaacetic acid (Gadovist, Bayer Healthcare, Leverkusen, Germany) administered using a contrast saline injector. Perfusion images were acquired in an end-expiratory breath-hold, with a high spatial resolution saturation recovery gradient echo method (repetition time/echo time 3.0ms/1.0ms, flip angle 15°, 5-fold *k-t* broad linear speed up technique (BLAST) acceleration and 11 training profiles, spatial resolution 1.2x1.2x10mm^3, 90 dynamic images). The mid-systolic slice, which was acquired in systole, was chosen for analysis as this cardiac phase allows the myocardial perfusion layers to be more easily separated.[1]

Heart rate and blood pressure were measured continuously throughout each protocol using a CMR compatible electrocardiogram and automated sphygmomanometer (set to inflate automatically every minute) respectively.

CMR Perfusion Analysis

Subendocardial and subepicardial contours were delineated for every dynamic of the perfusion series using dedicated software (EasyScil prototyping, Philips Medical Systems, Best, Netherlands). The software generated signal-intensity curves for the left ventricular blood pool and all myocardial segments, with myocardial signal-intensity values corresponding to relative contrast concentrations from the first-pass perfusion of Gadolinium. Perfusion estimates were obtained from signal-intensity

curves using a Fermi-function constrained deconvolution algorithm using Matlaboratory (MathWorks Inc., Natick, Massachusetts) as described by Jerosch-Herold et al.[2] This generated absolute baseline and stress myocardial blood flow values (ml/g/min) for all 6 segments of the mid-ventricular slice.

Transmural perfusion gradients (TPG) were obtained using dedicated software (EasyScil prototyping, Philips Medical Systems, Best, Netherlands). Using the same contours delineated for deconvolution analysis, subendocardial, $I_{endo}(\alpha,t)$, and subepicardial signal-intensity curves, $I_{epi}(\alpha,t)$, were obtained. Ten myocardial layers and sixty radial segments were sampled and a signal-intensity curve generated at each point. TPG curves $G(a,t)$ were calculated based on the difference in subendocardial and subepicardial signal-intensity values over time, t (each dynamic), at a particular myocardial location, α, and normalized to transmural signal-intensity values, $I_{transm}(\alpha,t)$.[3, 4] Peak intensity of the TPG was expressed as a percentage of transmural flow redistribution. A high-pass threshold of TPG was set at 5% to reduce the effect of noise of the measured perfusion gradients. The radial extent of the TPG in angular degrees (^{o}) was also calculated. A schematic representation of TPG analysis is shown in figure 2..

Cardiac Catheterization Laboratory Data Analysis

All pressure and flow signals were sampled at 200 Hz and stored on disk for off-line analysis. Ensemble averages of the selected cardiac cycles were performed for distal coronary artery pressure (P_d) and coronary blood flow (estimated with average peak Doppler flow velocity (APV)). Savitzky–Golay filters were applied to preserve peaks in the pressure and flow data while smoothing.[5] These filters fit a polynomial of a chosen order to a number of points around the centre point using least squares. They

have the advantage of smoothing data whilst preserving data peaks, which dramatically improves the clinical applicability of coronary wave intensity analyses. Net wave intensity (WI_{net}: $W.m^{-2}.s^{-2}$) was calculated to be the product of time derivatives (dt) of ensemble averaged and filtered coronary artery pressure (Pd) and flow (U) signals:[6]

$$\square I_{\square et} = \frac{\square Pd}{\square t} \square \frac{\square U}{\square t}$$

WI_{net} can be separated into forward contributing and backward components arriving at the measurement site:[7]

$$\square I_{\square et} = \square I_{+} + \square I_{-}$$

Where forward travelling waves (WI_{+}) are defined by a positive WI, which occurs when dP_d and dU change in the same direction. In contrast backward travelling waves (WI_) occur when dP_d and dU change in opposite directions, leading to a negative WI.

Waves are also defined as accelerating when dU increases, and decelerating when dU decreases. In addition waves can be defined as compression waves when associated with an increase in dP_d, or defined as expansion waves when associated with a decrease in dP_d. Four dominant waves were calculated: the accelerating forward compression wave (FCW); the decelerating forward expansion wave (FEW); the decelerating backward compression wave (BCW); and the accelerating backward expansion wave (BEW).[8] In the coronary circulation forward travelling waves are generated by increases (FCW) and decreases (FEW) in aortic pressure at the inlet and backward travelling waves are generated by changes in the microcirculation due to cardiac contraction (BCW) and relaxation (BEW). [9-11] Of these four waves, the acceleratory waves produced the greatest magnitude. Therefore only the two acceleratory waves, FCW and BEW, are reported in the manuscript.

In order to separate WI into the forward (WI_{+}) and backward (WI_) components these respective formulae were applied to collected data:

$$\square I_{+} = \frac{1}{4\rho\square}\left(\frac{\square P}{\square t} + \rho\square\frac{\square U}{\square t}\right)^{2}$$

$$\square I_{-} = -\frac{1}{4\rho\square}\left(\frac{\square P}{\square t} - \rho\square\frac{\square\square}{\square t}\right)^{2}$$

Where $\rho =$ density of blood, *c* = wavespeed, and WI was defined as the product of the first time derivatives of coronary artery pressure and flow velocity so that the analysis is independent of the sampling interval used, as previously described.[12] The density of blood was assumed to be constant at 1050kgm^{-3}.[7] Wavespeed was calculated using the single-point technique as previously described.[7]

Rate pressure product (RPP), a measure of myocardial oxygen demand,[13] was calculated as systolic blood pressure multiplied by heart rate. Diastolic time fraction was defined as the fraction of the duration of diastole with respect to duration of the cardiac cycle. Systolic duration was defined from the upslope of the arterial pressure trace to the dichrotic notch, and diastolic duration defined from the dichrotic notch to the upslope of the arterial pressure trace. Augmentation index, a measure of central systolic blood pressure augmentation thought to principally arise from pressure-wave reflection, was calculated as the difference between the first and second aortic systolic pressure peaks expressed as a percentage of the pulse pressure (in turn calculated as systolic blood pressure minus diastolic blood pressure).

Figure S1. Cardiac Catheterization Laboratory Protocol Set Up.

A. For cold air inhalation we adapted a clinical cryotherapy device with a closed cooling circuit: Cryo6 Cold Air Device (Zimmer Medizin Systeme, Ulm, Germany), at a fixed temperature of 5°F and a flow rate of 50L/min. A standard non-rebreathe facemask was adapted and fitted onto the end of the nozzle of the cryotherapy tubing. This therefore provided a means of supplying cold air for cold air inhalation.

B. Facemask application. The cold air chilled the cheeks, nose, mouth and chin, and mouth breathing was encouraged where possible (to avoid excess nasal warming of cold air).

C. An intracoronary 0.014-in guidewire (ComboWire, Volcano® Corporation, San Diego, USA) with Doppler velocity transducer on the wire tip and pressure transducer 1.5cm proximally to this on the wire shaft. This enabled simultaneous and continues measurement of coronary artery flow velocity and pressure. The ComboWire was inserted into the distal coronary artery (beyond the coronary artery stenosis in patients with coronary artery disease). When feasible the wire was inserted into the vessel retrogradely on a loop, which improved stability of the Doppler signal.

D. ComboMap console (Volcano® Corporation, San Diego, USA) demonstrating simultaneous acquisition of aortic pressure waveforms (red), distal coronary artery pressure waveforms (yellow) and Doppler velocity (blue with underlying grey scale) waveforms.

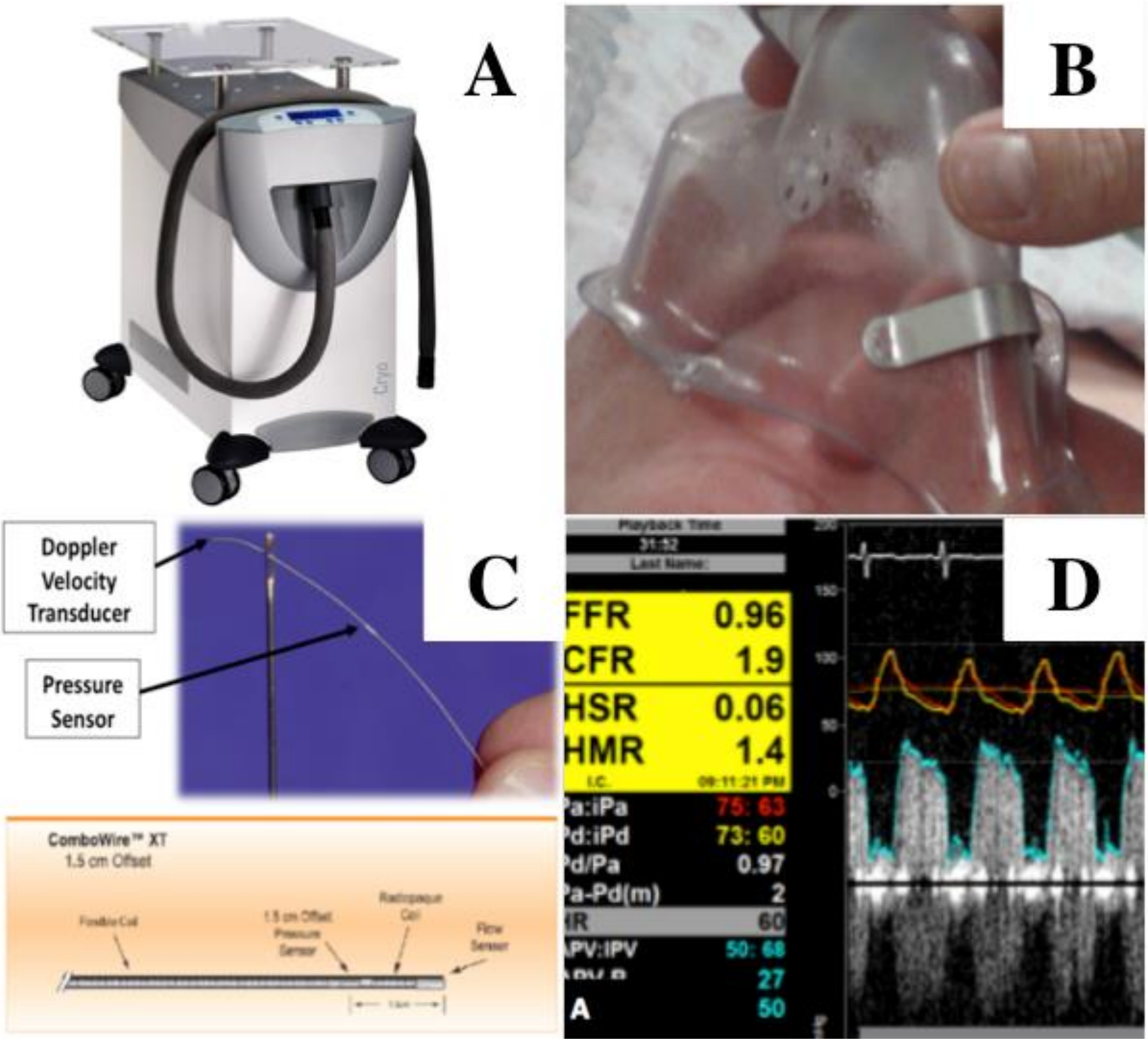
A
B
C
D
Doppler Velocity Transducer
Pressure Sensor
ComboWire™ XT
1.5 cm Offset
Playback Time
31:52
Last Name:
FFR 0.96
CFR 1.9
HSR 0.06
HMR 1.4
75: 63
73: 60
0.97
2
60
50: 68
27
50

Figure S2. Transmural Perfusion Gradients with CMR Perfusion Scans.

The grey scale images on the top half of this figure demonstrate typical high-resolution CMR perfusion images of three short-axis slices of the left ventricle. In this example there is visible subendocardial ischemia in the mid-ventricular and basal slices (shown as a darker rim of grey scale on the inside of the myocardium, highlighted with *). In order to quantify transmural perfusion gradients (TPG), subendocardial, subepicardial and left ventricular blood pool contours are drawn onto these images using EasyScil prototyping software. The TPG algorithm calculates the intensity of the gradient in each angular and temporal position by the spatial averaging of the signal intensity (of gadolinium contrast in the myocardium) of the inner and outer third of the left ventricular wall, normalised by the average transmural signal intensity to account for signal inhomogeneity. This creates a gradientogram plot that is shown on the lower half of this figure. The amplitude of the TPG is represented by the intensity of grey scale colour, so that a darker area represents a region of poorer subendocardial perfusion. A threshold TPG % can be set whereby an area above this set threshold represents a subendocardial perfusion deficit, as highlighted in green and highlighted with * (corresponding to the visual CMR perfusion images above). Temporal persistence in seconds (the length of time that the TPG is apparent for) is measured along the x-axis, and circumferential extent in angular degrees (the extent of myocardium involved) is measured along the y-axis. Reproduced in part from Chiribiri et al[4]with permission. Copyright ©2013, Elsevier.

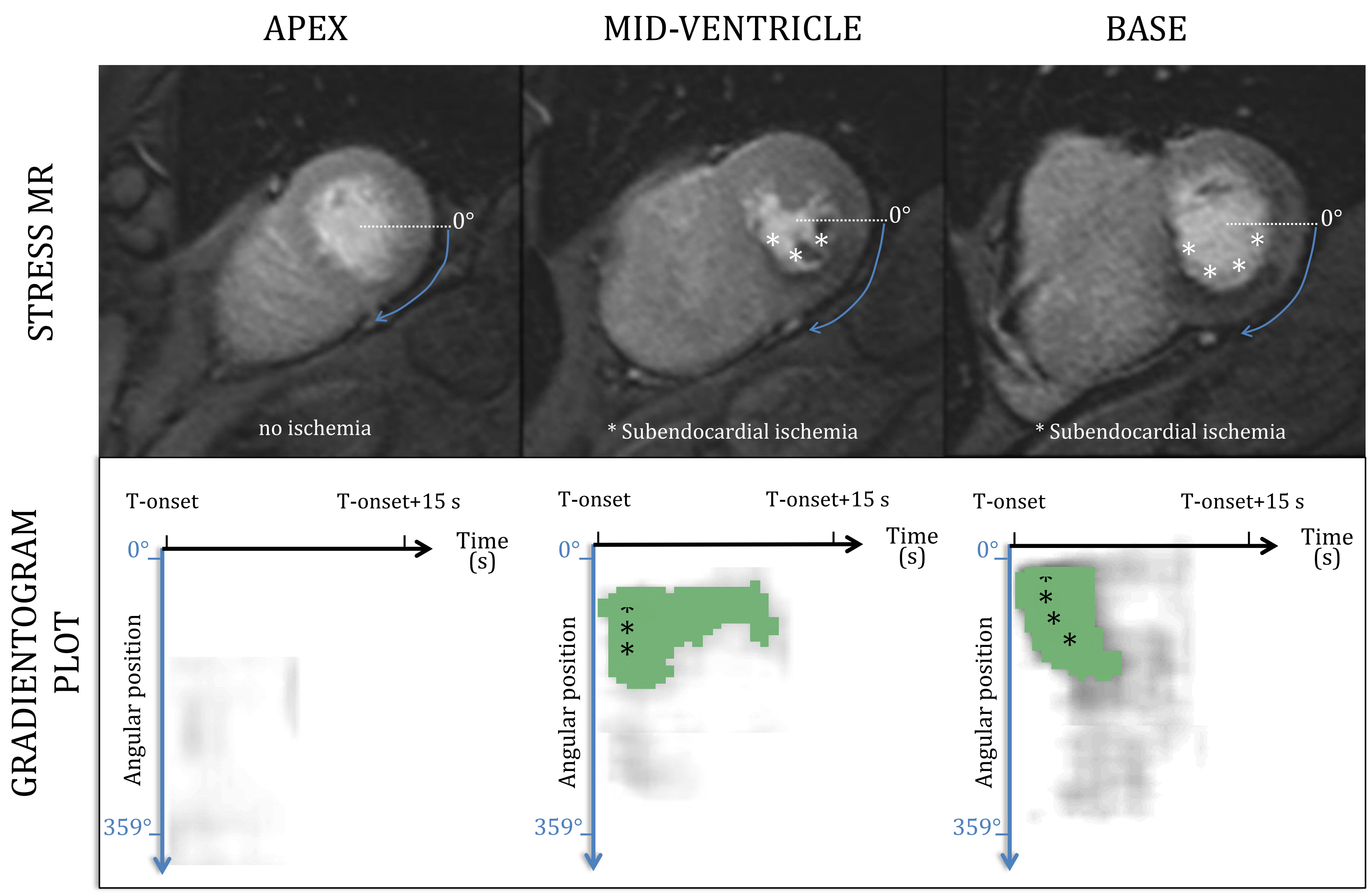
APEX
MID-VENTRICLE
BASE
STRESS MR
GRADIENTOGRAM PLOT
0°
no ischemia
* Subendocardial ischemia
* Subendocardial ischemia
T-onset
T-onset+15 s
Time (s)
Angular position
0°
359°

Figure S3. Transmural Perfusion Gradients during Cold Air Inhalation in Patients with Significant Coronary Artery Disease.

We observed significant increases in peak TPG intensity and radial TPG extent (both $p<0.05$). Peak TPG intensity reflects the subepicardial-subendocardial redistribution of myocardial blood flow, and radial extent TPG reflects the amount of myocardium affected. Increases in both parameters suggest an insufficiency of subendocardial perfusion. A peak TPG intensity value of greater than 20 % (during adenosine hyperemia) has been shown to strongly correlate with a significant fractional flow reserve (<0.8).[14]

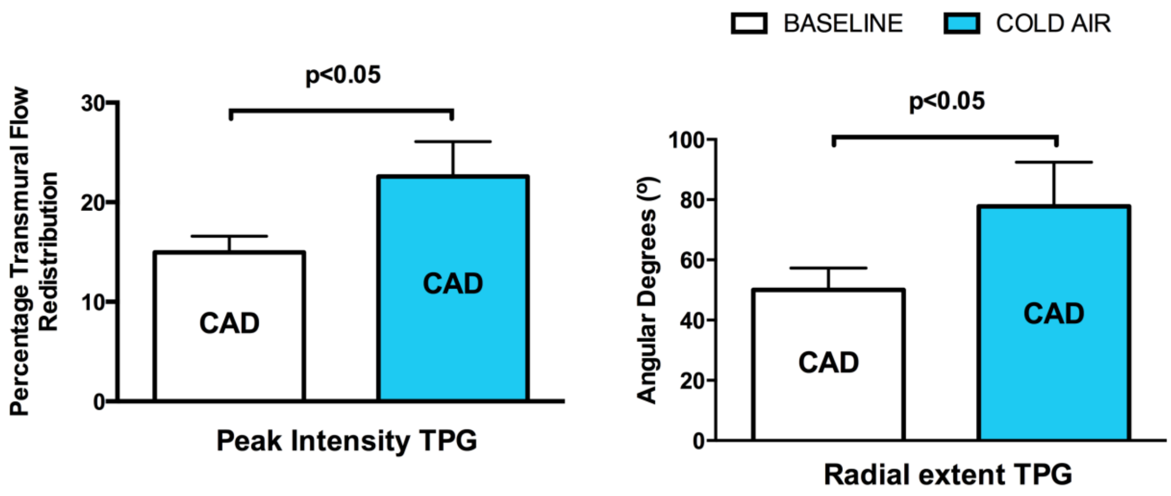

Supplemental Video Legend:

Video S1. Cardiac Catheterization Laboratory Protocol Set Up.

Supplemental References:

1. Fairbairn TA, Mather A, Larghat A, Greenwood J, Plein S. Measurement of myocardial blood flow response to the cold pressor test with myocardial perfusion CMR. *J Cardiovasc Magn Reson.* 2011;13:P312.
2. Jerosch-Herold M, Seethamraju RT, Swingen CM, Wilke NM, Stillman AE. Analysis of myocardial perfusion MRI. *J Magn Reson Imaging.* 2004;19:758-770.
3. Hautvast GL, Chiribiri A, Lockie T, Breeuwer M, Nagel E, Plein S. Quantitative analysis of transmural gradients in myocardial perfusion magnetic resonance images. *Magn Reson Med.* 2011;66:1477-1487.
4. Chiribiri A, Hautvast GL, Lockie T, Schuster A, Bigalke B, Olivotti L, Redwood SR, Breeuwer M, Plein S, Nagel E. Assessment of coronary artery stenosis severity and location: quantitative analysis of transmural perfusion gradients by high-resolution MRI versus FFR. *JACC Cardiovasc Imaging.* 2013;6:600-609.
5. Steinier J, Termonia Y, Deltour J. Smoothing and differentiation of data by simplified least square procedure. *Anal Chem.* 1972;44:1906-1909.
6. Parker KH. An introduction to wave intensity analysis. *Med Biol Eng Comput.* 2009;47:175-188.
7. Davies JE, Whinnett ZI, Francis DP, Willson K, Foale RA, Malik IS, Hughes AD, Parker KH, Mayet J. Use of simultaneous pressure and velocity measurements to estimate arterial wave speed at a single site in humans. *Am J Physiol Heart Circ Physiol.* 2006;290:H878-85.

8. Bleasdale RA, Parker KH, Jones CJ. Chasing the wave. Unfashionable but important new concepts in arterial wave travel. *Am J Physiol Heart Circ Physiol.* 2003;284:H1879-85.

9. Sun YH, Anderson TJ, Parker KH, Tyberg JV. Wave-intensity analysis: a new approach to coronary hemodynamics. *J Appl Physiol.* 2000;89:1636-1644.

10. Davies JE, Whinnett ZI, Francis DP, Manisty CH, Aguado-Sierra J, Willson K, Foale RA, Malik IS, Hughes AD, Parker KH, Mayet J. Evidence of a dominant backward-propagating "suction" wave responsible for diastolic coronary filling in humans, attenuated in left ventricular hypertrophy. *Circulation.* 2006;113:1768-1778.

11. Spaan J, Kolyva C, van den Wijngaard J, ter Wee R, van Horssen P, Piek J, Siebes M. Coronary structure and perfusion in health and disease. *Philos Trans A Math Phys Eng Sci.* 2008;366:3137-3153.

12. Niki K, Sugawara M, Chang D, Harada A, Okada T, Sakai R, Uchida K, Tanaka R, Mumford CE. A new noninvasive measurement system for wave intensity: evaluation of carotid arterial wave intensity and reproducibility. *Heart Vessels.* 2002;17:12-21.

13. Kitamura K, Jorgensen CR, Gobel FL, Taylor HL, Wang Y. Hemodynamic correlates of myocardial oxygen consumption during upright exercise. *J Appl Physiol.* 1972;32:516-522.

14. Tonino PA, De Bruyne B, Pijls NH, Siebert U, Ikeno F, van' t Veer M, Klauss V, Manoharan G, Engstrom T, Oldroyd KG, Ver Lee PN, MacCarthy PA, Fearon WF. Fractional flow reserve versus angiography for guiding percutaneous coronary intervention. *N Engl J Med.* 2009;360:213-224.
